# Primary aorto enteric fistula a rare but life threatening condition: a case report

**DOI:** 10.4076/1757-1626-2-6821

**Published:** 2009-09-14

**Authors:** TL Luk

**Affiliations:** 1 Department of Vascular Surgery, Royal Bournemouth Hospital, Castle Lane East, Bournemouth, Dorset, BH7 7DW, UK

## Abstract

Primary aorto enteric fistula is a very rare but life threatening condition. We report a case of primary aorto enteric fistula in a 62-year-old man whose diagnosis was only made at laparotomy. A high index of suspicion along with a good history and physical exam is critical for making a successful diagnosis. Surgical exploration is warranted if other investigation is not conclusive.

## Introduction

Aorto enteric fistula (AEF) is a rare life threatening complication of aortic surgery. It occurs in 0.3-4% of patients who underwent open AAA repair. Primary fistula formation between the aorta and the duodenum is however considerably rarer with an incidence rate at autopsy of 0.04% to 0.07%. We report a case of primary AEF in a 62-year-old man whose diagnosis was only made at laparotomy.

## Case presentation

**Figure 1:**
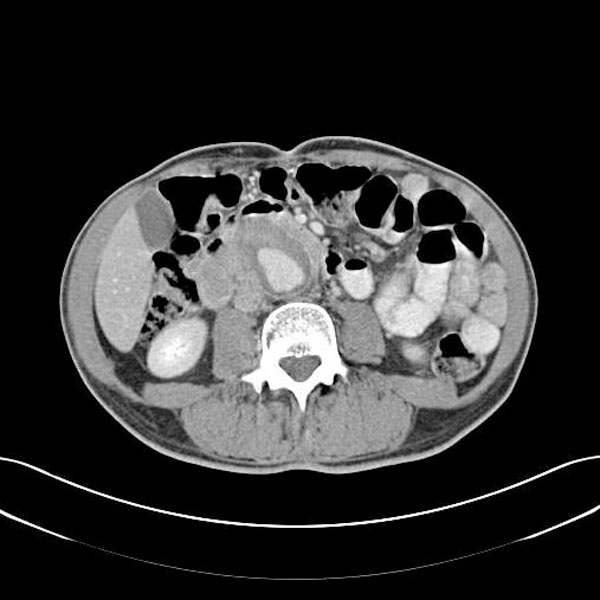
**CT showing AAA**.

This 62-year-old British Caucasian man presented to general surgery with a 2 weeks history of non specific right sided abdominal pain and 10 kg of weight loss over the last 6 months. Blood tests including FBC, renal function and electrolytes were normal. Abdominal ultrasound scan did not reveal any intra abdominal pathology. The aorta was measured at 2.6 cm. This pain settled with oral analgesia and he was discharged home with plan for further investigation as an out patient. Six days later the patient was readmitted with right sided abdominal pain and rectal bleeding. Patient has a normal pulse rate, blood pressure and was non febrile. Examination revealed a soft abdomen, palpable aorta and tenderness to the right of the umbilicus. Digital rectal examination revealed small amount of blood on the glove. Blood test showed a haemoglobin level of 12.4 g/dL, elevated WCC of 21 × 10^9^/L and a CRP of 21 mg/L. He has a strong history of ischaemic heart disease with three previous episode of myocardial infarction, the last one being over one year ago. An urgent CT scan of the abdomen and pelvis were organised to rule out a malignant cause of abdominal pain. Patient has no further episode of rectal bleeding and clinically appeared well. The following day, patient collapsed while walking to the toilet. His pulse rate was 110/min with a systolic pressure of 80 mmHg. CT scan early in the day showed a 5 cm infrarenal AAA. (Figure 1)

Patient was taken to theatre with a presumed diagnosis of ruptured aneurysm. The on call surgeon who happened to be an upper GI surgeon and an experienced vascular surgeon performed the operation. At laparotomy, patient was found to have an inflamed aorta with a fistula communicating with the 3^rd^ part of the duodenum. The aorta was debrided and repaired using a Dacron tube graft and wrapped in collatamp sheet. This was further covered using an omentum pedicle brought through the transverse mesocolon separating the Dacron graft from the duodenum. The duodenal defect was primarily closed. Patient made good recovery and was discharged on day 8 post surgery with long term antibiotic. Patient remained well at 6 months follow up.

## Discussion

Primary AEF is exceedingly rare with fewer than 200 cases reported worldwide. It is first reported by Sir Ashley Cooper in 1822 [1]. The proposed theories for the formation of primary ADF are direct wear and inflammatory destruction triggered by infection, foreign bodies or erosions. The characteristic site of formation is the 3^rd^ or 4^th^ part of the duodenum, the fixed retroperitoneal portion just anterior to the aorta. They usually occur in the setting of a large AAA abutting the bowel with fistula formation over time. 73% were from atherosclerotic aneurysms and 26% were from traumatic or mycotic aneurysms. Other causes include radiation, metastases, pancreatic carcinoma, ulcers, gallstones, diverticulitis, appendicitis and cystic medial necrosis [2]. In this particular case, the culture has grown Coliforms. The infectious agents involved in mycotic aneurysms are most commonly salmonella or Klebsiella. Other agents like tuberculosis, syphilis, mycosis, staphylococcus and streptococcus can also be responsible [3].

The most common clinical signs and symptoms of AEF are upper or lower gastrointestinal bleeding (64%), abdominal pain (32%) and a pulsatile abdominal mass (25%) [4]. Most patients are seen with an intermittent herald bleed that presented as melaena with relatively few haemodynamic consequences. This can be followed by a catastrophic bleeding and exsanguination. Herald bleeds occur as clot fills the fistula with subsequent small bowel contraction. This bleed can occur hours to several days before diagnosis is made. Other symptoms may include upper abdominal pain, back pain, fever and sepsis. Physical examination may demonstrate palpable AAA with abdominal bruit.

In this case the initial abdominal ultrasound scan measured the aorta at 2.6 cm. By definition, this is not aneusymal. The subsequent CT scan measured it at 5 cm. It is difficult to explain the large discrepancy of size. It could be due to operator error or it could be rapid expansion of the anerusym following the initial scan. Also there is always the slight discrepancy in measurement of size between ultrasound and CT. If the initial scan has picked up an AAA, a CT would certainly follow rapidly as the patient presented with abdominal pain. This might help to reach the diagnosis earlier.

AEF should be considered in all patients with GI bleeding and a history of AAA or previous aortic revascularisation with prosthetic graft. A high index of suspicion is the key. Although diagnostic tests cannot entirely eliminate the possibility of AEF, endoscopy is commonly employed followed by CT scan and angiography. Over 90% of AEF are within the reach of endoscopy. Also endoscopy are usually easily accessible in most hospital and can exclude other causes of upper GI bleed. With the continual improvement in CT imaging, it has fast becoming the preferred diagnostic test especially if endoscopy failed to demonstrate the cause. Loss of the aneurysmal wall or the fat plane between the aorta and the duodenum or air in the retroperitoneum is highly suggestive of AEF. Angiography defines arterial anatomy and may be useful especially in patients with previous aortic reconstruction. However very few primary AEF are diagnosed with angiography.

Exploratory laparotomy is indicated for patients with suspected AEF and unremitting gastrointestinal bleeding. The mortality rate of untreated AEF is 100%. Surgical intervention is mandatory for survival and successful outcome. With appropriate treatment, survival rates varying from 18% to 93% has been reported [2]. Post operative complications occur in as many as 40% of cases. Primary AEF associated with infected AAAs has a worse prognosis than primary AEF associated with AAA alone, with post operative mortality rate exceeding 50% [3].

Surgical repair of the AAA and fistula is the standard treatment. In cases of primary AEF with minimal infection, anatomic in situ AAA repair with Dacron and primary repair of the bowel and placement of omentum between the bowel and the prosthetic graft is preferred. In cases of gross infection and contamination, extensive debridement and extra anatomic bypass graft is advocated. Harvesting the patient's superficial femoral vein and using it as graft or specially prepared homograft has also been used in infected cases. In this case, collatamp sheet which are gentamicin impreganated collagen sponge was used to wrap around the Dacron graft followed by placement of omentum acting as a barrier to prevent infection. Intra operative cultures should be taken and antibiotic treatment tailored to the culture and sensitivities. If cultures are negative, empiric antibiotics to cover the most likely contaminating organism are usually recommended for 7-10 days.

## Conclusion

Primary AEF is a very rare cause of GI bleeding and abdominal pain. However mortality rate is high if the diagnosis is missed. A high index of suspicion along with good history and physical exam is critical for making a successful diagnosis. Surgical exploration is warranted with continued bleeding and high index of suspicion even if OGD and CT scan is not diagnostic.

## Abbreviations

AAA: abdominal aortic anerusym; AEF: aorto enteric fistula; GI: gastro intestinal; OGD: oesophagogastroduodenoscopy.

## Consent

Written informed consent was obtained from the patient for publication of this case report and accompanying images. A copy of the written consent is available for review by the Editor-in-chief of this journal.

## Competing interests

The author declares that they have no no competing interests.
